# First report of the genus *Rhyncholagena* Lang, 1944 from the South China Sea, with the description of a new species (Crustacea, Copepoda, Harpacticoida, Miraciidae)

**DOI:** 10.3897/zookeys.805.24331

**Published:** 2018-12-11

**Authors:** Lin Ma, Xin-zheng Li

**Affiliations:** 1 Laboratory of Marine Organism Taxonomy and Phylogeny, Institute of Oceanology, Chinese Academy of Sciences, Qingdao 266071, China Institute of Oceanology, Chinese Academy of Sciences Qingdao China; 2 Center for Ocean Mega-Science, Chinese Academy of Sciences, Qingdao, 266071, China Center for Ocean Mega-Science, Chinese Academy of Sciences Qingdao China; 3 Graduate University, University of Chinese Academy of Sciences, Beijing 100039, China Graduate University, University of Chinese Academy of Sciences Beijing China

**Keywords:** Benthic copepods, Crustacea, new species, taxonomy

## Abstract

During analysis of sediment samples from South China Sea, a new species belonging to the genus *Rhyncholagena* Lang, 1944 was found and described here. *Rhyncholagenaparaspinifer***sp. n.** differs from its congeners by the following combined characteristics: body ornamented dorsally with at least one row of spinules on each somite except penultimate urosomite; A2 exopod two-segmented; P1 enp-2 with one inner seta; P3 exp-3 with two inner setae, P3 enp-2 with one inner seta; female P5 exopod with five setae; male P5 baseoendopod with two setae and exopod with four setae. This is the first report of the genus *Rhyncholagena* in the China seas. In addition, a key to all valid species of *Rhyncholagena* is given, along with tables of morphological characters of all valid species and their distributions.

## Introduction

The harpacticoid genus *Rhyncholagena* Lang, 1944 belongs to the large family Miraciidae Dana, 1846, comprising nine species and subspecies (Walter et al. 2015), plus the new species described here. The genus *Rhyncholagena* was erected to accomodate three species previously assigned to *Amphiascus* Sars G.O., 1905. The genus *Rhyncholagena* was distinguished from *Amphiascus* by subtle morphological characteristic such as rostrum flask shape (Lang 1948). Por (1967) thought that the main characters of the genus were the incision between the apical setae of the P5 exopod and the bottle-like or elongated rostrum.

Species of the genus *Rhyncholagena* are benthic forms that inhabit different marine environments: gravels (Por 1964), mud (Present contribution), sand (Sars 1911), mangrove (Malt 1990), and coral reefs (Sarmento and Santos 2012). Members of this genus range from intertidal to subtidal areas of continental shelves (Lang 1948; Por 1964, 1967; Malt 1990). Species of *Rhyncholagena* were reported from different regions of world: *R.lagenirostris* (Sars G.O., 1911) was from Norway; *R.spinifer* (Farran, 1913) from Ireland and France; *R.pestaipestai* (Monard, 1935) from France, Algeria and North Carolina of the USA; *R.pestaiamericana* Rouch, 1962 from Argentina; *R.levantina* Por, 1964 from coasts of Israel and France in Mediterranean; *R.josaphatis* Por, 1967 from the Red Sea and Suez Canal; *R.littoralis* Por, 1967 from the Red Sea, Suez Canal and Brazil; *R.profundorum* Por, 1967 from the Red Sea; and *R.bermudensis* Malt, 1990 from Bermuda.

The South China Sea is a semi-enclosed marginal sea of the tropical Indo-Pacific region. The knowledge about the composition and distribution of benthic harpacticoids are considered as insufficient (Chertoprud et al. 2011). During a survey of the local macrobenthos along the coasts of Hainan Island in the South China Sea, we took some sediment samples from subtidal of east Hainan Island. Harpacticoid copepods were sorted from these samples. A new species of *Rhyncholagena* was found and is described here. This is the first report of the genus from the South China Sea. Finally, data about the depth and sample locality of all valid species were collected to discuss the distribution of the genus *Rhyncholagena* and a worldwide identification key to species is provided.

## Materials and methods

Sediment samples were collected from the South China Sea, fixed in 10% formalin. Sediment samples were washed through a 38 μm sieve with tap water. The harpacticoid specimens were extracted from remaining sediment samples by centrifugation with the colloidal silica Ludox TM-50 suspension as flotation medium. Specimens were preserved in 75% alcohol. For their identification, the specimens were cleared in lactic acid and observed with a light microscope. Before dissection, the habitus was drawn and the whole body length was measured temporarily mounted in lactophenol. Specimens were dissected in lactic acid and mounted on slides in lactophenol, subsequently sealed with nail-polish. The observations and drawings were made with a differential interference contrast microscope (Nikon Eclipse Ni), equipped with a drawing tube. The illustration of habitus were drawn at 400× magnification, the others were drawn at 1000× magnification, with oil immersion lens.

The terminology used is after Huys et al. (1996). Abbreviations used in the text and figures are:

**A2** antenna;

**aes** aesthetasc;

**exp** exopod;

**exp-1 (-2-3)** the first (second, third) segment of the exopod;

**enp** endopod;

**enp-1 (-2-3)** the first (second, third) segment of the endopod;

**P1–P6** swimming legs 1–6.

Body length was measured from the anterior margin of the rostrum to the posterior margin of the caudal rami. The type material is deposited in the Marine Biological Museum, Chinese Academy of Sciences, Qingdao, China (**MBMCAS**).

## Results

### Order Harpacticoida Sars, 1903

#### Family Miraciidae Dana, 1846

##### Subfamily Diosaccinae Sars, 1906

###### Genus *Rhyncholagena* Lang, 1944

####### Rhyncholagena paraspinifer sp. n.

Taxon classificationAnimaliaORDOFAMILIA

http://zoobank.org/E4E9C696-1020-47DF-960B-E6ADE07C2C6B

Figs 1 , 2 , 3 , 4 , 5 , 6 , 7 , 8

######## Type locality.

South China Sea, sampling locality (18°35.81'N, 110°43.44'E), 30.1 m depth, soft mud, collected by JB Wang, LM Shuai, J Zhou, QX Han and L Ma, 19 October 2007.

######## Material examined.

Holotype 1♀ dissected on three slides (MBM189117). Paratypes: 1♀ on one slide (MBM189079), 1♂ (MBM189080) on one slide and 6 ♀♀, 4 ♂♂ (MBM189081) in 70 % ethanol. Allotype1 ♂ on two slides (MBM189118). All paratypes and allotype were collected from the type locality.

######## Description.

*Female* (based on holotype and one paratype).

**Figure 1.**
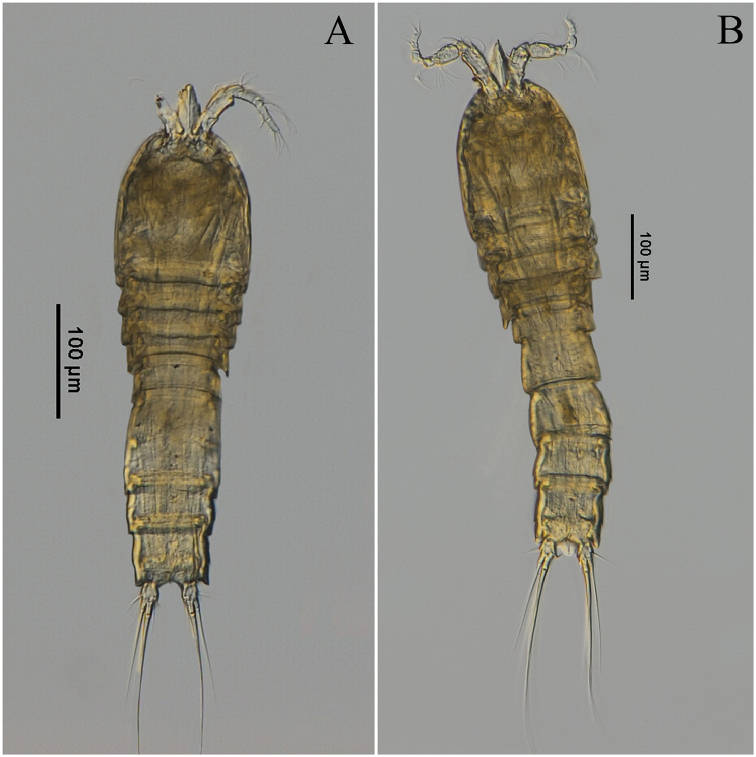
*Rhyncholagenaparaspinifer* sp. n. **A** Paratype (female,MBM189079) habitus, dorsal **B** Paratype (male, MBM189080) habitus, dorsal.

**Figure 2.**
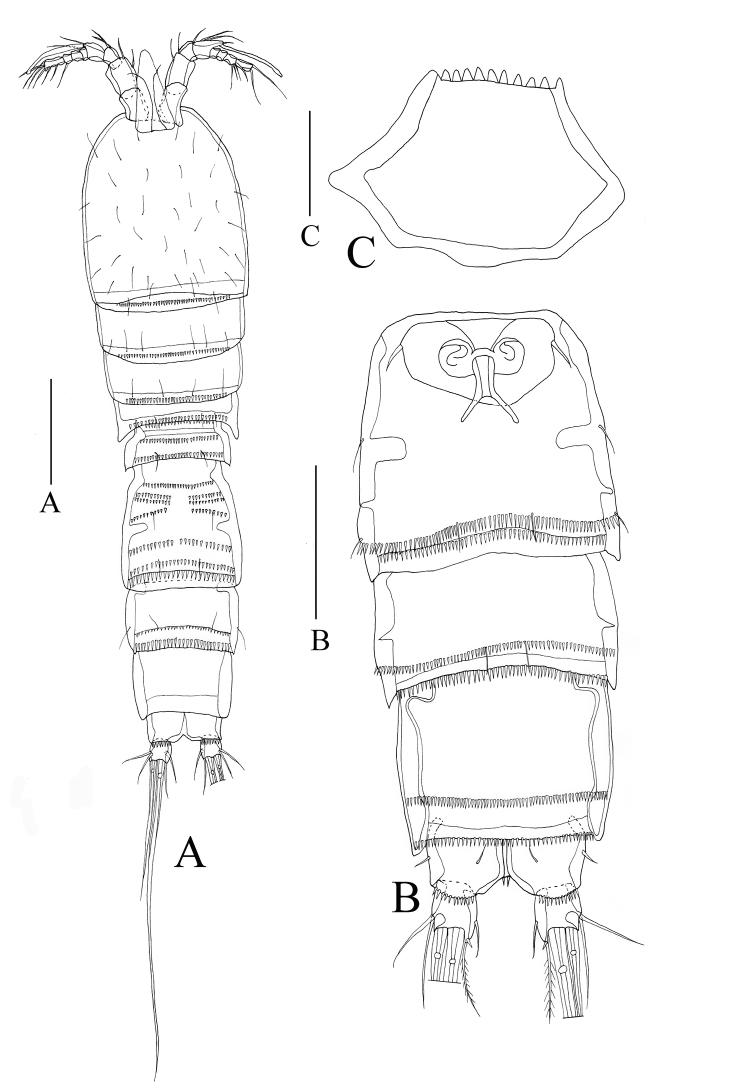
*Rhyncholagenaparaspinifer* sp. n. Holotype **A** habitus, dorsal **B** urosome, ventral **C** labrum. Scale bars: 100μm (**A, B**); 50μm(**C**).

*Habitus* (Figs 1A, 2A, B). Total length of holotype female (body plus caudal rami, excluding caudal setae): 710µm. Body long and cylindrical, widest at head, tapering posteriorly. Prosome four-segmented: cephalothorax (including two thoracic somites bearing maxilliped and P1) and three articulated somites bearing P2 to P4; all prosomites with row of spinules on posterior margins, respectively. Urosome five-segmented, comprising P5-bearing somite, genital double-somite, and three abdominal somites. Urosomites with rows of hyaline frills on dorsal edge respectively, excluding penultimate urosomite; urosomites ornamented with hyaline frills on ventral side. Genital field (Figure 2B) located rather proximally, genital apertures situated ventrally, covered by reduced P6 on both sides. Anal somite slightly cleft in posterior, unornamented, 0.4 times as long as wide; anal operculum narrow, unornamented. Caudal ramus almost as long as broad, carrying six setae: two outer setae, smooth; two medial setae, well developed; two inner setae, slender.

Rostrum (Figs 2A, 4A) demarcated from cephalothorax, elongated, almost triangular with pair of sensillae on each side of rostrum approx. 1/3 from acute tip.

Labrum (Figure 2C) somewhat hexagonal, with toothed fringe at tip.

**Figure 3.**
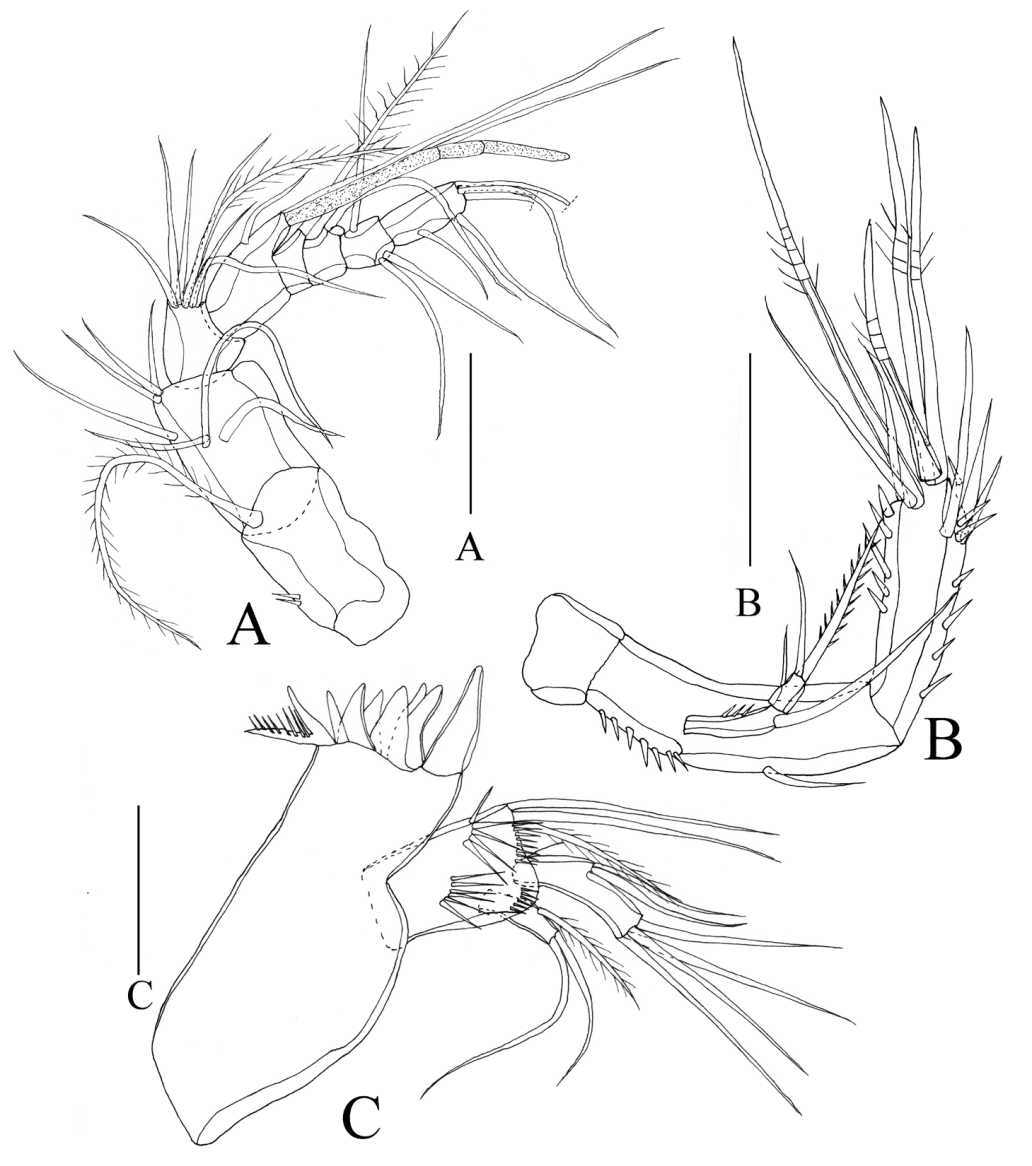
*Rhyncholagenaparaspinifer* sp. n. Holotype **A** antennule **B** antenna; paratype (female, MBM189079) **C** mandible. Scale bar: 50 μm.

Antennule (Figure 3A) with eight segments; first segment and second segment the longest; aesthetasc on fourth segment reaching beyond distal end of terminal segment. Armature formula: 1-[1], 2-[7], 3-[7], 4-[3+aes], 5-[1], 6-[1], 7-[3], 8-[5].

Antenna (Figure 3B) biramous, small coxa without ornamentation. Allobasis elongated, about three times as long as coxa, with spinules on lateral margin. Exopod two-segmented, with 1:1.2 setae; exp-1 long, almost two times as long as exp-2. Endopod one-segmented, with row of spines on inner and out edge, respectively; lateral armature consisting of three smooth setae; apical armature consisting of six elements: four geniculate setae, two slender and smooth setae.

Mandible (Figure 3C) gnathobase with eight large, smooth teeth and one seta, outmost teeth combined with seta on the base. Basis with four rows of spinules and three setae. Exopod one-segmented, with one lateral seta, two terminal setae. Endopod one-segmented, with two lateral setae, four terminal setae.

**Figure 4.**
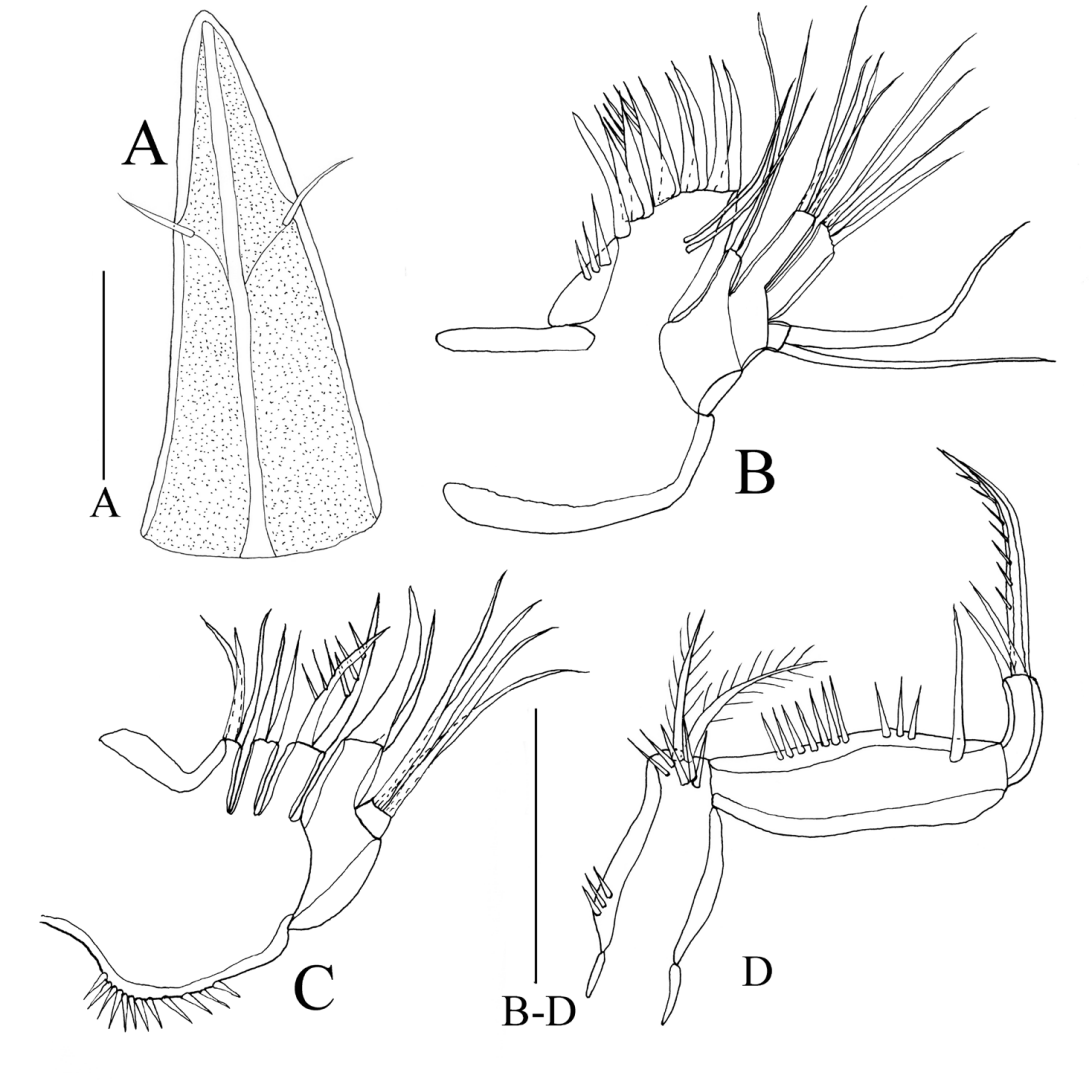
*Rhyncholagenaparaspinifer* sp. n. Holotype **A** rostrum **B** maxillule **C** maxilla **D** maxilliped. Scale bar: 50 μm.

Maxillule (Figure. 4B). Praecoxa and coxa demarcated. Arthrite with nine apical spines, two juxtaposed setae on surface. Coxal endite with two setae. Basis with four naked setae. Endopod one-segmented, with four naked setae. Exopod one-segmented, with two setae.

Maxilla (Figure 4C). Syncoxa with spinules along outer margin; with three endites bearing three, two, two setae, respectively. Allobasis with one claw and one seta. Endopod one-segmented, with four setae, respectively.

Maxilliped (Figure 4D). Subchelate. Syncoxa with several spinules along inner and distal margins, two setae located at distal margin. Basis with row of spinules and one seta on inner margin. Endopod one-segmented; with two setae and one strong claw.

**Figure 5.**
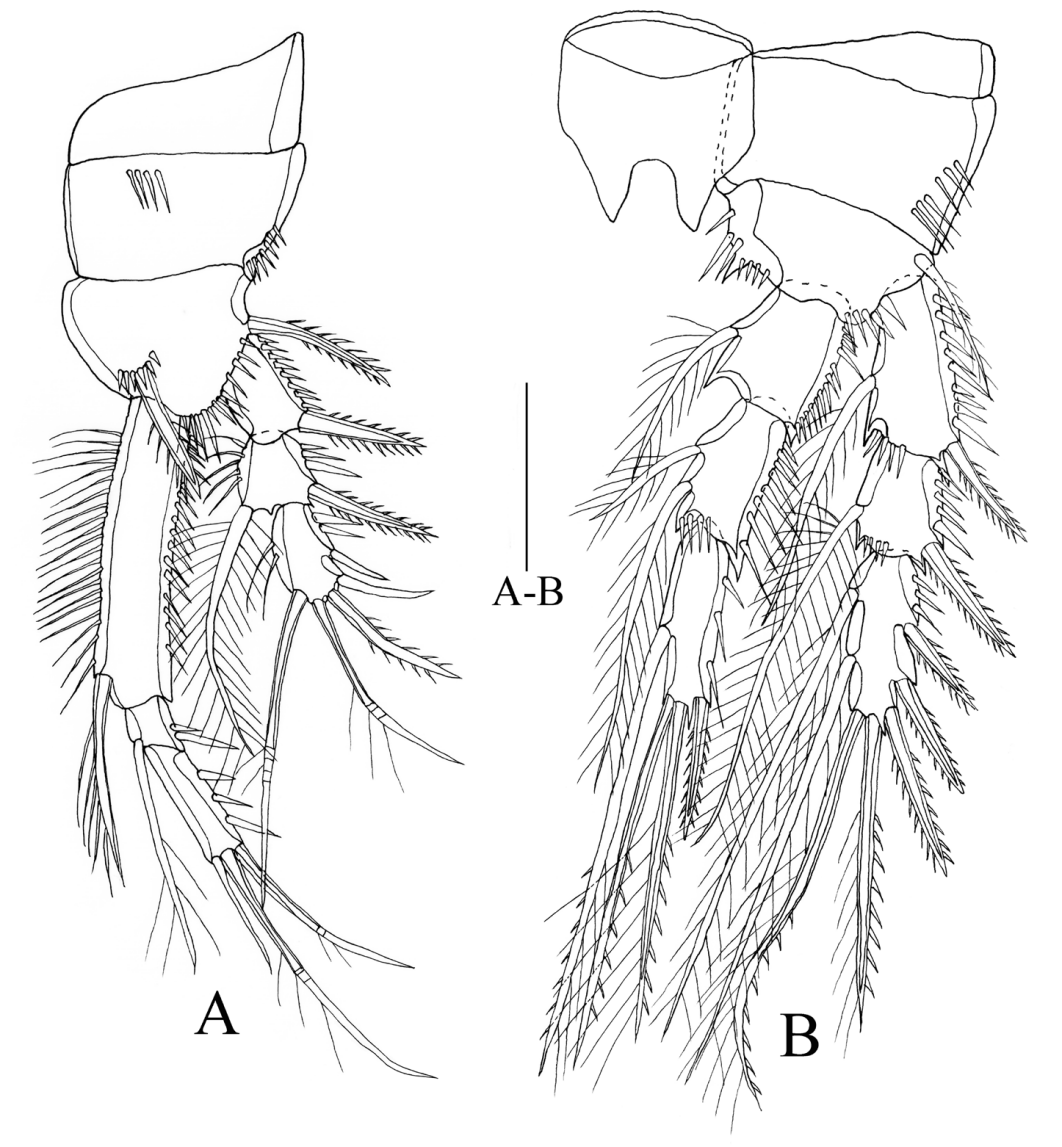
*Rhyncholagenaparaspinifer* sp. n. Holotype **A** P1, anterior **B** P2, anterior. Scale bar: 50 μm.

P1 (Figure 5A). Coxa with row of spines on anterior surface, row of spinules along outer margin. Basis bearing one outer plumose seta and one strong inner spinulose spine, terminal margin with spinules, surface with setules. Exopod three-segmented, short, reaching to nearly 4/5 length of enp-1; outer margins of each segment ornamented with spinules; inner margins of exp-2 and exp-3 with setules; exp-2 with one plumose seta; exp-3 with two geniculate setae, two spinulose spines and one smooth spine. Endopod three-segmented, outer margins of each segment with spinules;enp-1 elongated, 1.8 times as long as enp-2 plus enp-3, inner margin with setules and one plumose seta; enp-2 short, with one inner seta; enp-3 longer than exp-2, approx. twice as long as enp-2.

**Figure 6.**
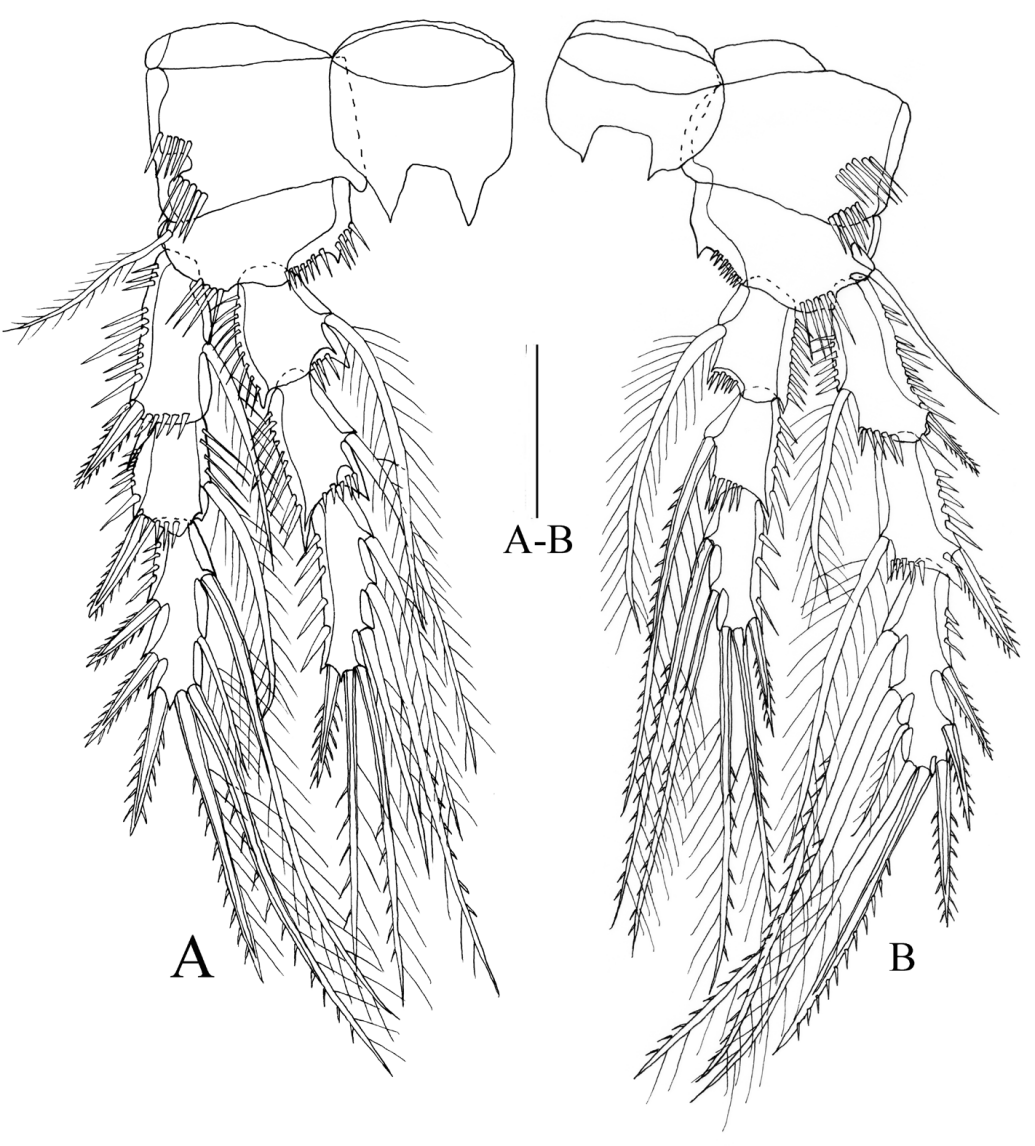
*Rhyncholagenaparaspinifer* sp. n. Holotype **A** P3, anterior **B** P4, anterior. Scale bar: 50 μm.

P2–P3 (Figs 5B, 6A). Intercoxal sclerites approximately triangular, with two distal (or apical) projections. Coxae with row of spines on outer margins. Basis with rows of spines on anterior margins. Exopods and endopods three-segmented, outer margins of each segment ornamented with spinules; endopod nearly as long as exopod.

P4 (Figure 6B) Intercoxal sclerites almost quadrate, with two distal blunt projections. Coxa of almost rectangular shape with two rows of spinules on anterior surface. Basis with row of spines on anterior margin. Exopods and endopods three-segmented, endopod shorter than exopod. Setal formulae of female P1–P4 as follows:

**Table:** 

	**Exp**	**Enp**
P1	0–1–1, 2, 2	1–1–0, 2, 1
P2	1–1–2, 2, 3	1–2–1, 2, 1
P3	1–1–2, 2, 3	1–1–3, 2, 1
P4	1–1–3, 2, 3	1–1–2, 2, 1

**Figure 7.**
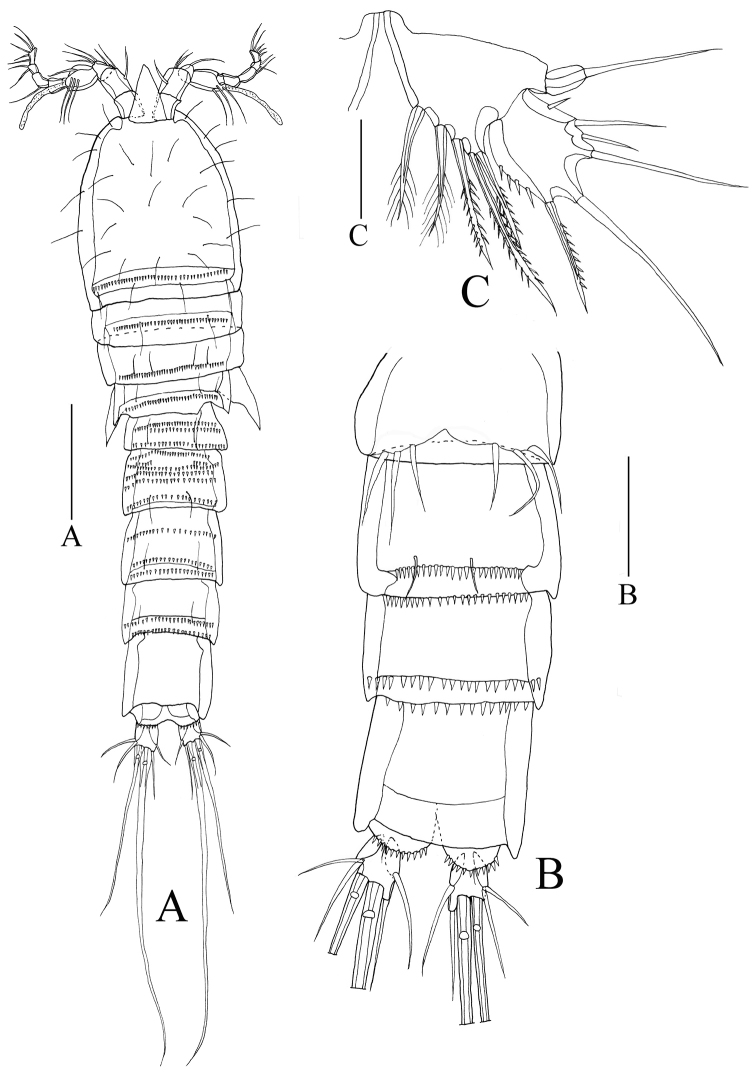
*Rhyncholagenaparaspinifer* sp. n. Allotype **A** habitus, dorsal **B** urosome, ventral; holotype **C** P5. Scale bars: 100 μm(**A, B**); 50 μm(**C**).

Right and left P5 (Figure 7C) not fused medially, baseoendopod and exopod separated. Baseoendopod reaching nearly to 1/5 length of exopod; with two plumose and three spinulose setae, second innermost one longer than others. Exopod nearly rectangular, 1.3 times as long as greatest width, ornamented with one spinulose and four naked setae, distalmost one longest.

*Male* based on allotype and one paratype differs from female as follows:

Body (Figs 1B, 7A) slightly shorter than female holotype, total length of allotype male (body plus caudal rami, excluding caudal setae): 610 µm. Urosome (Figure 7B) six-segmented, genital somite and the first abdominal somite separate, urosomites with rows of small spinules except penultimate urosomite. Caudal ramus as long as broad, with six setae.

**Figure 8.**
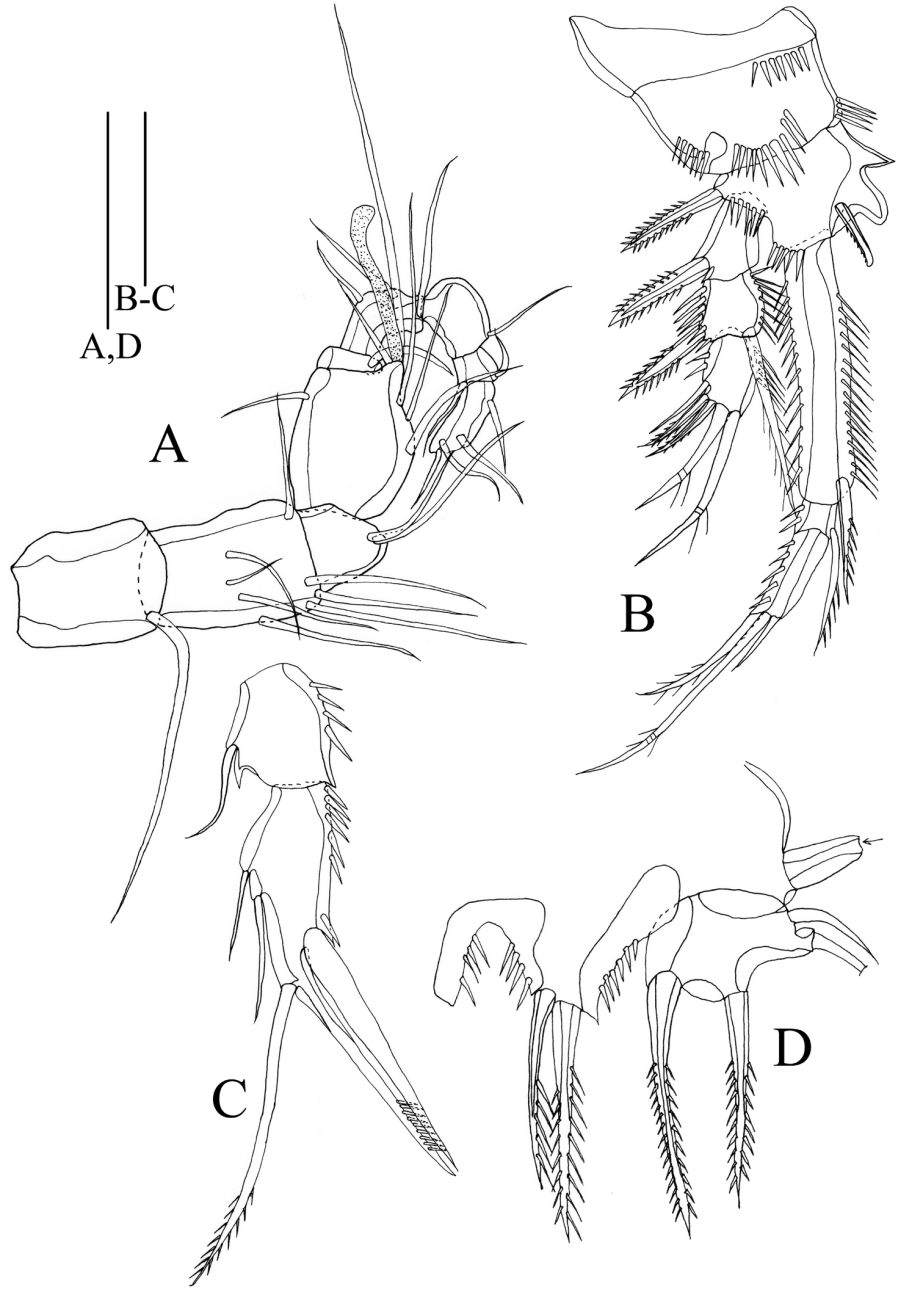
*Rhyncholagenaparaspinifer* sp. n. Allotype **A** antennule **B** P1, anterior **C** P2 endopod, anterior **D** P5 with one outer seta cutting; paratype (male, MBM189080; small arrow meaning one seta missing). Scale bars: 50 μm.

Antennule (Figure 8A) nine-segmented, haplocer. Armature formula: 1-[1], 2-[8], 3-[2], 4-[6+aes], 5-[1], 6-[3], 7-[1], 8-[2], 9-[4], geniculation between sixth and seventh segments.

Antenna, mandible, maxillule, maxilla, maxilliped, P3 and P4 similar to female.

P1 (Figure 8B). Coxa with four rows of spines on anterior surface and inner margin. Basis bearing one fingerlike spine and two spinous projections on inner margin; exp-1 without spines on outer margin. Other characters as in female.

P2 with protopod and exopod as in female holotype. Endopod (Figure 8C) two-segmented; enp-1 with one slender inner seta; enp-2 modified as common in genus, with two slender setae on inner margin; one seta and one spinous spine on distal margin; one stout spine on outer margin.

P5 (Figure 8D) baseoendopod unseparated, with two spinous spines, reaching beyond the end of the exopod; exopod with denticles and four unequal setae, two pinnate, two slender and naked.

P6 (Figure 7B) reduced each to three setae inserted on distal margin of somite.

######## Variability.

Most morphological features are conservative, except body length. Body length of female varies from 450µm to 710µm and male from 460 µm to 610 µm.

######## Etymology.

The species is named according to many spines on the body.

## Discussion

The new species can be easily placed in the genus *Rhyncholagena* by the following two characters: the incision between the apical setae of the P5 exopod and the very elongated rostrum (see Por 1967).

In Table 1, all currently valid species of *Rhyncholagena* are listed, together with some of their most prominent morphological characters. All morphological characters were collected from publications, except for the new species described here. Some original species descriptions were not thorough, and additional data are collected from subsequent publications attributed to the same species.

**Table 1.** List of valid species of *Rhyncholagena* Lang, 1944, with their most prominent morphological features according original descriptions and additional data about the same species.

Species	A2 exopod segment	Setal formulae of swimming legs (exp/enp)				Setal formulae of swimming legs (exp/enp)		Female P5 exopod long/wide	Distal of female P5 baseoendopod	References
		P1	P2	P3	P4	female P5	male P5			
*R.bermudensis* Malt, 1990	2	0.1.122/1.1.021	1.1.223/1.2.130	1.1.223(2)/ 1.1.231	1.1.323/1.1.230	6 /5	Unknown	≈3	not exceeding half-length of exopod	Malt 1990
*R.josaphatis* Por, 1967	3	0.1.122/1.0.120	1.1.223/1.2.121	1.1.223/1.2.321	1.1.323/1.1.221	6 /4	5 (2)	≈1.9	exceeding half-length of exopod	Por 1967
*R.lagenirostris* (Sars, 1911)	3	0.1.122/1.1.120	1.1.123/1.2.121	1.1.123/1.1.321	1.1.223/1.1.221	6 /5	5 (3)	≈2.4	slightly exceeding half-length of exopod	Sars 1911; Lang 1948; Jakobi 1959; Wells 2007
*R.levantina* Por, 1964	Unknown	0.1.122/1.1.120	1.1.123/1.1.121	1.1.123/1.1.321	1.1.323/1.1.221	5 /5	5 (2)	≈2.2	exceeding half-length of exopod	Por 1964
*R.littoralis* Por, 1967	3	0.1.122/1.0.120	1.1.223/1.2.121	1.1.223/1.1.321	1.1.323/1.1.221	6/5	Unknown	≈1.6	not exceeding half-length of exopod	Por 1967
*R.pestaipestai* (Monard, 1935)^a^	3	0.1.122/1.1.120	1.1.223/1.2.121	1.1.223/1.2.321	1.1.323/1.1.221	6/5	6(3)	≈2.1	slightly exceeding half-length of exopod	Monard 1935; Lang 1948; Jakobi 1959; Wells 2007
*R.pestaiamericana* Rouch, 1962	3	0.1.122/1.1.120	1.1.223/1.2.121	1.1.223/1.1.321	1.1.323/1.1.221	6/5	Unknown	≈2.5	exceeding half-length of exopod	Rouch 1962
*R.profundorum* Por, 1967	3	0.1.122/1.1.120	1.1.223/1.2.121	1.1.223/1.2.321	1.1.323/1.1.221	5/5	Unknown	≈1.9	not exceeding half-length of exopod	Por 1967
*R.spinifer* (Farran, 1913)	3	0.1.122/1.0.120	1.1.123/1.2.121	1.1.123/1.1.321	1.1.223/1.1.221	6/5	5 (3)	≈2.9	slightly exceeding half-length of exopod	Farran 1913; Lang 1948; Jakobi 1959; Wells 2007
*R.paraspinifer* sp. n.	2	0.1.122/1.1.120	1.1.223/1.2.121	1.1.223/1.1.321	1.1.323/1.1.221	5/5	4(2)	≈1.2	not exceeding half-length of exopod	Present contribution

^a^Bodin (1964) mentioned that caudal ramus of the species were not twisted inside as type species, but bulbous-shaped; exopod of male P5 with five setae instead of six.

It is clear from Table 1, that the species of genus *Rhyncholagena* can be separated into two groups based on the number of inner seta in P1 enp-2. The group 1 without inner seta on P1 enp-2, includes *R.spinifer*, *R.josaphatis*, *R.littoralis*. The group 2 with one inner seta on P1 enp-2, comprises *R.lagenirostris*, *R.pestaipestai*, *R.pestaiamericana*, *R.levantina*, *R.profundorum*, *R.bermudensis* and *R.paraspinifer* sp. n. Within this genus, *R.pestaipestai* may be a doubtful species. Monard (1935) described the species with the caudal ramus bulbous-shaped and twisted inside; exopod of male P5 with six setae. Bodin (1964) mentioned the caudal ramus of the species as bulbous-shaped, but not twisted inside as in type species; and the exopod of male P5 with five setae. We can’t know if the differences can be attributed to intraspecific variability or can be an error of observation. The new species differs from its congeners by the combined morphological features: body ornamented with hyaline frills except penultimate urosomite on distal margin; A2 exopod two-segmented; P1 enp-2 with one inner seta; P3 exp-3 with two inner setae; P3 enp-2 with one inner seta; female P5 exopod with five setae; male P5 baseoendopod with two setae, exopod with four setae. The shape of P5 is another particular character useful to differentiate the *Rhyncholagena* species. According to our observations, P5 of our specimens resembles those of *R.littoralis* Por, 1967 and *R.profundorum* Por, 1967. These three species share the following characters in the female P5: distal of P5 baseoendopod not exceeding half-length of exopod; P5 exopod less than twice as long as wide; projection of two apical setae in exopod nearly the same length.

However, *R.paraspinifer* sp. n. differs from *R.littoralis* by the following characteristics: rostrum almost triangular (needle-like in *R.littoralis*); A2 exopod two-segmented (three-segmented in *R.littoralis*); P1 enp-2 with one inner seta (without inner seta in *R.littoralis*); P5 exopod bearing five setae (six setae in *R.littoralis*). *Rhyncholagenaparaspinifer* sp. n. can be distinguished from *R.profundorum* by the following features: A2 exopod two-segmented (three-segmented in *R.profundorum*); P3 enp-2 with one inner seta (two inner setae in *R.profundorum*); P5 exopod 1.15 times as long as wide (1.85 times in *R.profundorum*); P5 exopod nearly rectangular (oval in *R.profundorum*).

*Rhyncholagenaparaspinifer* sp. n. bears two inner setae in P3 exp-3, in contrast to *R.lagenirostris*, *R.levantina*, and *R.spinifer* which bear only one inner seta. However, *R.paraspinifer* sp. n. differs from *R.josaphatis* and *R.pestaipestai* by having two and four setae in male P5 baseoendopod and exopod, respectively (two and five setae in *R.josaphatis*; three and six setae in *R.pestaipestai*). The new species can be distinguished from *R.bermudensis* by the characters of the two apical projections of P5 exopod being as long as each other in female (the longer one nearly twice as long as shorter one in *R.bermudensis*). *Rhyncholagenaparaspinifer* sp. n. and *R.pestaiamericana* shares similar setal formulae of P1-P4. However, the two species also have differences: *R.paraspinifer* sp. n. bears five setae on female P5 exopod (six setae in *R.pestaiamericana*); distal of P5 baseoendopod not exceeding half-length of exopod in female (exceeding half-length of exopod in *R.pestaiamericana*).

The distributions and depths of all valid species of the genus *Rhyncholagena* are listed in Table 2. From the Table 2, we can consider that the genus *Rhyncholagena* is mainly distributed in the Atlantic Ocean, the Red Sea, and the Mediterranean and ranges from intertidal to deep sea. No cosmopolitan species were found. The recent record from the South China Sea considerably extended the distribution range of the *Rhyncholagena* species to Indo-Pacific Ocean. The fact that most species of *Rhyncholagena* were reported in single localities only, does not indicate that they are endemic. The low number of known *Rhyncholagena* species from the Pacific Ocean maybe due to lack of sampling of this taxon. More samples have to be analyzed to gain more knowledge about the distribution of the taxon *Rhyncholagena*.

From the Table 2, we can found that species of the genus *Rhyncholagena* are mainly distributed in temperate zone, except *R.paraspinifer* in subtropical zone, *R.littoralis* in temperate and tropical zone. It is interesting to note that the species *R.littoralis* is eurythermal and inhabits in both sides of Atlantic. More studies would be necessary to elucidate the distribution of the species.

**Table 2.** The distributions of valid species of genus *Rhyncholagena* Lang, 1944.

Species	Distribution	Depth	References
*R.bermudensis* Malt, 1990	Bermuda (mangrove)	9–11m	Malt 1990; Warwick et al. 1990
*R.josaphatis* Por, 1967	Red Sea; Suez Canal	5–300m	Por 1967, 1977; Por and Marcus 1972
*R.lagenirostris* (Sars, 1911)	Norway	36.58–54.87m	Sars 1911; Lang 1948
*R.levantina* Por, 1964	Nahariya (Israel); Banyuls Sur Mer (France)	3m	Por 1964; Guille and Soyer 1966
*R.littoralis* Por, 1967	Red Sea; Suez Canal; Brazil (coral reefs)	0.5–1m(gravels)	Por 1967; Por and Marcus 1972; Sarmento and Santos 2012
*R.pestaipestai* (Monard, 1935)	France (Roscoff; North Brittany); Algeria (Castiglione);North Carolina (USA); France (Marseilles)	10–30m	Monard 1935, 1937; Lang 1948;Coull 1971; Bodin 1964; Bodin and Le Guellec 1992
*R.pestaiamericana* Rouch, 1962	Argentina (Punta Canteras)	250m	Rouch 1962
*R.profundorum* Por, 1967	Red Sea	700m	Por 1967
*R.spinifer* (Farran, 1913)	Ireland (Killary harbour in Mayo); France (North Brittany)	43.9m	Farran 1913; Monard 1928; Bodin and Le Guellec 1992
*R.paraspinifer* sp. n.	South China Sea	30.1m	Present contribution

Lang (1948) established a key to species of the genus *Rhyncholagena*, which included three species, *R.lagenirostris*, *R.spinifer* and *R.pestaipestai*. Below we present an updated key, which is modified from the earlier keys by Lang (1948) and Wells (2007). Since some species lack descriptions of male, the key is made based on females.

### Key to the species of the genus *Rhyncholagena* Lang, 1944 (female)

**Table:** 

1	P5 baseoendopod with four setae	*** R. josaphatis *** **Por, 1967**
–	P5 baseoendopod with five setae	**2**
2	P5 exopod with five setae	**3**
–	P5 exopod with six setae	**5**
3	P3 enp-2 with two inner setae	***R.profundorum*** **Por, 1967**
–	P3 enp-2 with one inner seta	**4**
4	P2 enp-2 with two inner setae	*** R. paraspinifer *** **sp. n.**
–	P2 enp-2 with one inner seta	***R.levantina*** **Por, 1964**
5	P2-P3 exp-3 with one inner seta	**6**
–	P2-P3 exp-3 with two inner setae	**7**
6	Urosomites without hyaline frills; second segment of A1 produced a well-marked and incurved spinous projection in middle inside	***R.lagenirostris*** **(Sars G.O., 1911)**
–	Urosomites with 8 strong and separated spines on posterior dorsal margins; second segment of A1 without spinous projection in middle inside	*** R. spinifer *** ** (Farran, 1913)**
7	P2 enp-2 with two inner setae	***R.pestaipestai*** **(Monard, 1935)**
–	P3 enp-2 with one inner seta	**8**
8	P5 exopod with two apical projections, longer one about two times as long as shorter one	***R. bermudensis*** **Malt, 1990**
–	Two apical projections of P5 exopod mostly as long as each other	**9**
9	P1 exopod not exceeding to middle length of P1 enp-1; P5 exopod less than two times as long as greatest wide	***R.littoralis*** **Por, 1967**
–	P1 exopod exceeding to middle length of P1 enp-1; P5 exopod more than two times as long as greatest wide	***R.pestaiamericana*** **Rouch, 1962 **

## Supplementary Material

XML Treatment for Rhyncholagena paraspinifer
